# CodLncScape Provides a Self‐Enriching Framework for the Systematic Collection and Exploration of Coding LncRNAs

**DOI:** 10.1002/advs.202400009

**Published:** 2024-04-11

**Authors:** Tianyuan Liu, Huiyuan Qiao, Zixu Wang, Xinyan Yang, Xianrun Pan, Yu Yang, Xiucai Ye, Tetsuya Sakurai, Hao Lin, Yang Zhang

**Affiliations:** ^1^ Tsukuba Life Science Innovation Program University of Tsukuba Tsukuba 3058577 Japan; ^2^ Innovative Institute of Chinese Medicine and Pharmacy Academy for Interdiscipline Chengdu University of Traditional Chinese Medicine Chengdu 611137 China; ^3^ Department of Computer Science University of Tsukuba Tsukuba 3058577 Japan; ^4^ Department of Developmental Biology School of Basic Medical Sciences Southern Medical University Guangzhou 510515 China; ^5^ School of Healthcare Technology Chengdu Neusoft University Chengdu 611844 China; ^6^ School of Life Science and Technology University of Electronic Science and Technology of China Chengdu 611731 China

**Keywords:** coding lncRNAs, computational biology, computational precision health, data collection, machine learning

## Abstract

Recent studies have revealed that numerous lncRNAs can translate proteins under specific conditions, performing diverse biological functions, thus termed coding lncRNAs. Their comprehensive landscape, however, remains elusive due to this field's preliminary and dispersed nature. This study introduces codLncScape, a framework for coding lncRNA exploration consisting of codLncDB, codLncFlow, codLncWeb, and codLncNLP. Specifically, it contains a manually compiled knowledge base, codLncDB, encompassing 353 coding lncRNA entries validated by experiments. Building upon codLncDB, codLncFlow investigates the expression characteristics of these lncRNAs and their diagnostic potential in the pan‐cancer context, alongside their association with spermatogenesis. Furthermore, codLncWeb emerges as a platform for storing, browsing, and accessing knowledge concerning coding lncRNAs within various programming environments. Finally, codLncNLP serves as a knowledge‐mining tool to enhance the timely content inclusion and updates within codLncDB. In summary, this study offers a well‐functioning, content‐rich ecosystem for coding lncRNA research, aiming to accelerate systematic studies in this field.

## Graphical abstract

codLncScape consists of a set of interconnected tools that work together to create a self‐sustaining loop of knowledge expansion in coding lncRNA research. The cycle begins with codLncDB, moves on to codLncFlow, and then to codLncWeb, which leads to codLncNLP, before returning to codLncDB. Specifically, it includes a knowledge base, analysis workflow, online platform, and NLP model.

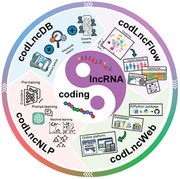

## 1 Introduction

Long non‐coding RNAs (lncRNAs), a key class of RNA molecules widely distributed across the genome, are traditionally not considered to have protein‐coding capabilities. Nonetheless, they are involved in various fundamental biological processes, such as signal transduction, post‐transcriptional modification, and the formation of cellular structure, thereby playing an indispensable role in regulating gene expression and maintaining cellular functionality.^[^ ^1^ ^]^ Compared to other RNA molecules, lncRNAs are characterized by their structural and functional diversity, complex genomic locations, and generally low levels of endogenous expression. These attributes intensify the challenge of deciphering lncRNA functions.^[^ ^1^, ^2^ ^]^

Recent studies have progressively revealed that certain lncRNAs contain open reading frames (ORFs) and, under specific spatiotemporal conditions, can be recognized by cellular translation mechanisms, resulting in the synthesis of short peptides or proteins. These molecules play roles in a variety of physiological and pathological processes, including cellular differentiation, tissue development, and tumor formation.^[^ ^3^ ^]^ Such lncRNAs with coding potential are referred to as coding lncRNAs. For instance, as non‐coding capacity, LINC00961 reduces β‐catenin protein levels in Non‐Small Cell Lung Cancer (NSCLC), thereby inhibiting cellular invasion and metastasis.^[^ ^4^ ^]^ LINC00961 could also code the peptide SPAR and interact with lysosomal v‐ATPase to negatively regulate mTORC1 activation.^[^ ^3b^ ^]^ Similarly, LINC00673 enhances the interaction between PTPN11 and PRPF19, an E3 ubiquitin ligase, thereby diminishing SRC‐ERK oncogenic signaling in pancreatic cancer.^[^ ^5^ ^]^ Except for its non‐coding capacity, LINC00673 could code a novel peptide, RASON, which serves as an enhancer of oncogenic RAS signaling.^[^ ^6^ ^]^ The widespread existence of translation events in lncRNAs has been further substantiated by techniques like ribosome profiling, high‐throughput CRISPR screening, and mass spectrometry.^[^ ^7^ ^]^ Taken together, the discovery of coding lncRNAs not only adds a new dimension to our understanding of the genomic coding landscape and its regulatory complexity but also blurs the distinction between coding and non‐coding RNAs, prompting researchers to reconsider RNA functions and evolution.^[^ ^8^ ^]^

Currently, the exploration of translational events in lncRNAs and the identification of novel coding lncRNAs have emerged as focal areas in lncRNA research.^[^ ^7^, ^9^ ^]^ However, most of the individual coding lncRNA studies, while valuable, often only reveal a fraction of the real coding lncRNA universe. This limited scope has led to a gap in comprehensive and systematic research of coding lncRNAs, potentially leading to biases in our overall understanding of this field. To mitigate this issue, several studies have begun to aggregate coding lncRNAs and establish related databases, such as ncEP,^[^ ^10^ ^]^ FuncPEP,^[^ ^11^ ^]^ EVLncRNAs 2.0,^[^ ^12^ ^]^ cncRNAdb,^[^ ^8a^ ^]^ and LncPep.^[^ ^13^ ^]^ Nevertheless, these databases contain a limited number of high‐confidence coding lncRNA entries. Alongside these efforts, researchers also attempted to systematically identify endogenously translated peptides from coding lncRNAs, but functional investigations in this area are still limited.^[^ ^14^ ^]^

In light of the scarcity of systematic studies, we introduce codLncScape, a well‐functioning and self‐enriching framework containing an interconnected suite of resources and tools for coding lncRNA exploration. At its foundation, codLncScape incorporates codLncDB, a manually compiled knowledge base of coding lncRNAs. Leveraging codLncDB, we then conducted codLncFlow, a computational workflow of coding lncRNA to explore their implications in pathological and physiological conditions. Further enhancing its utility, we developed codLncWeb, a platform for storing, browsing, and retrieving data across various programming environments. Finally, we established a codLncNLP, a knowledge‐mining tool that integrated natural language processing (NLP) models, such as prompt‐learning ChatGPT‐4, to enhance the timely inclusion and update of the content in codLncDB. This initiative is aimed at constructing a rich, scalable ecosystem focused on coding lncRNAs, with the intention of accelerating research in this area and reducing knowledge biases.

## 2 Results

### 2.1 The Overview of codLncScape

**Figure 1:**
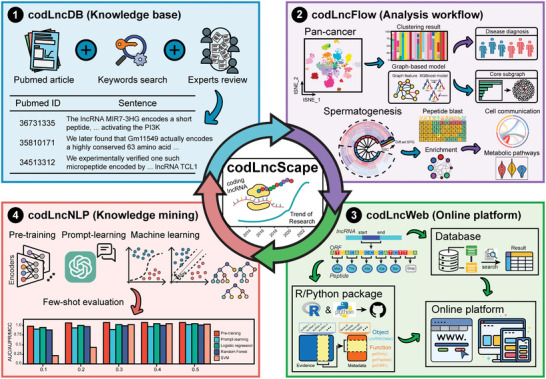
Schematic illustration of codLncScape. The first component, codLncDB, was established through a process of keyword‐driven searches in PubMed literature, complemented by manual reviews to extract low‐throughput validation data for coding lncRNAs. The second component, codLncFlow, was a workflow leveraging data from codLncDB to conduct unsupervised clustering and follow‐up analysis in the context of pan‐cancer and spermatogenesis. The third component, codLncWeb, consisted of a database and R/Python modules available for third‐party, all hosted on an accessible online platform. The final component, codLncNLP, integrated a prompt‐learning model with pre‐training and machine‐learning models, offering a robust toolkit for future data retrieval and update.

The general architecture of codLncScape encompassed 4 main components: the establishment of a coding lncRNA knowledge base (codLncDB), the exploration of coding lncRNAs in pathological (pan‐cancer) and physiological (spermatogenesis) contexts (codLncFlow), the development of a platform for storing, browsing, and fetching data (codLncWeb), and the establishment of a knowledge‐mining tool (codLncNLP) for timely content update (**Figure** **1**). Fundamental to our study is the creation of a self‐sustaining cycle of research that continually propels the field forward. From codLncDB to codLncFlow, through codLncWeb, to codLncNLP, and back to codLncDB, this cycle ensures ongoing contributions to lncRNA research.

Specifically, our initial step involved filtering articles from PubMed using keywords, supplemented by rigorous expert review, to create codLncDB, a manually curated knowledge‐base of 353 coding lncRNA entries (supported by low‐throughput experiments). Notably, 293 of these entries represent novel additions not cataloged in other databases (Figure S1, Supporting Information; Experimental Section). Our subsequent focus was to elucidate the implications of these coding lncRNAs in various contexts, including pathological and physiological conditions. We conducted a global coding lncRNA‐centric clustering analysis and then examined their diagnostic potential in pan‐cancer contexts. Alongside, we explored the differentiation‐associated coding lncRNAs and tried to infer their functions during spermatogenesis. Then, to facilitate the widespread data analysis, we developed an online platform, codLncWeb, embedding database, R, and Python packages for storing, browsing, and fetching coding lncRNAs data. This platform facilitates research across various programming environments. Finally, we incorporated an array of machine‐learning approaches, a pre‐training model,^[^ ^15^ ^]^ and a ChatGPT 4.0‐based^[^ ^16^ ^]^ prompt‐learning model to establish a knowledge‐mining tool and thereby ensure the continuous updating and expansion of the content in codLncDB.

### 2.2 Decoding the Role of Coding LncRNAs Across Diverse Cancers

**Figure 2:**
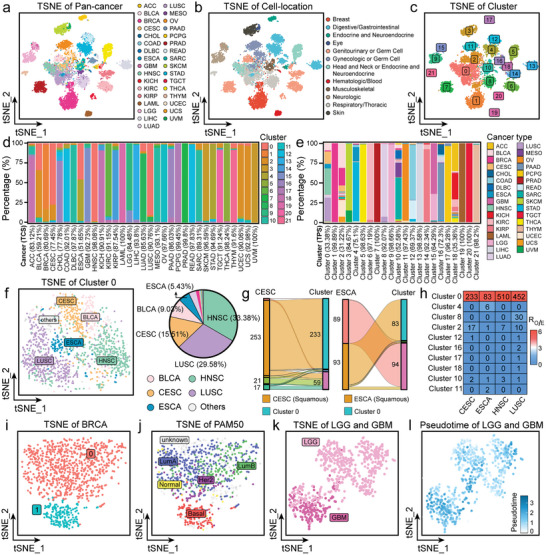
Decoding the role of coding lncRNAs in pan‐cancer. a–c) t‐SNE visualization of pan‐cancer RNA‐seq data for coding lncRNAs (9,784 samples). Samples were colored by a) cancer sources, b) cell origin, and c) clustering results. d) The bar charts showed the distribution of clusters among the cancer types. Each bar indicated tumor clustering score (TCS). e) The bar charts showed the distribution of cancer types among the clusters. Each bar indicated the cluster purity score (TPS). (f) t‐SNE visualization of cluster 0, with samples colored by cancer type. The pie chart showed the proportions of cancer types in cluster 0. g) Sankey plots showed the distribution of squamous cell carcinoma samples from CESC and ESCA within different clusters. h) The cluster enrichment of each squamous cell carcinoma sample was estimated by Ro/e (ratio of observed and expected samples). The heatmap displayed the number of observed samples and was colored by Ro/e values. i) t‐SNE visualization of BRCA samples, colored by clustering result and j) molecular subtype (PAM50Call_RNAseq). t‐SNE visualization of LGG and GBM samples, colored by k) cancer types and l) pseudotime.

Numerous studies have elucidated the significant correlation between coding lncRNAs and their translated micropeptides with the onset and progression of various cancers.^[^ ^17^ ^]^ Thus, we systematically analyzed the expression of coding lncRNAs across diverse cancers and their relationship with cancer pathology. A total of 140 coding lncRNAs were detected in the TCGA pan‐cancer dataset (9,784 samples from 33 cancer types). Upon performing dimension reduction analysis (t‐SNE) based on the 140 coding lncRNAs, we observed an obvious cancer‐type preference, as depicted in **Figures** **2**a and S2 (Supporting Information). Consistent with previous findings,^[^ ^18^ ^]^ we found that tumor samples sharing the same cellular origin also tended to cluster together (Figure 2b). Consequently, we employed an unsupervised graph‐based clustering algorithm, which identified 22 distinct clusters among the tumor samples, denoted as cluster 0 to cluster 21 (Figure 2c). To elucidate the associations between these 22 clusters and the 33 cancer types, we formulated two evaluative metrics: The Tumor Clustering Score (TCS) represents whether a certain type of cancer tends to cluster together, while the Tumor Purity Score (TPS) indicates whether the samples within a cluster are likely to originate from a specific type of cancer. Our analysis revealed that 75% of cancer types achieved high TCS values (>86.03%) (Figure 2d; Figure S3a, Supporting Information). This finding underscored the potent efficacy of coding lncRNAs in tumor classification. Meanwhile, though 75% of clusters exhibited high TPS values (>70.10%), such as clusters 1, 5, 6, 20, and 21 (Figure 2e; Figure S3b, Supporting Information), others, like clusters 0, 2, and 18, displayed low TPS values (<50%). This disparity implied that certain clusters contain samples from multiple cancer types.

Subsequently, we focused on clusters exhibiting low TPS. Specifically, we scrutinized cluster 0, which comprised samples from various tumor types, such as Head and Neck Squamous Cell Carcinoma (HNSC), Lung Squamous Cell Carcinoma (LUSC), Cervical Squamous Cell Carcinoma (CESC), Bladder Urothelial Carcinoma (BLCA), and Esophageal Carcinoma (ESCA), as indicated in Figure 2f. Notably, over half of the samples in this cluster were from HNSC (33.38%) and LUSC (29.58%), both representing squamous cell carcinomas. This led us to speculate whether cluster 0 tended to aggregate various types of squamous cell carcinomas. Further analysis of the CESC and ESCA samples within this cluster substantiated our hypothesis, revealing that 233 out of 253 squamous cell samples from CESC and 83 out of 93 from ESCA were clustering in cluster 0 (Figure 2g). Furthermore, we quantified the enrichment of squamous cell samples in each cluster based on the ratio of observed to expected values (*R*_*O*/*E*_), as illustrated in Figures 2h and S3c (Supporting Information). The results demonstrated significant enrichment of squamous cell samples from HNSC, LUSC, CESC, and ESCA in cluster 0. These findings implied that cluster 0 had a lower TPS, since it predominantly aggregated squamous cell carcinoma samples from different cancers, indirectly highlighting the ability of coding lncRNAs in identifying squamous cell carcinomas regardless of cancer origin.

Moreover, we extended our analysis to examine the capacity of coding lncRNAs for subtyping certain cancers. For instance, in the case of Breast Cancer (BRCA), we observed a distinct division of samples into two separate clusters: 82.79% (909 out of 1098) of the samples were categorized into cluster 0, while 17.21% (189 out of 1098) were allocated to cluster 1, as shown in Figure 2i. Clinical annotations based on the PAM50 molecular subtyping of BRCA elucidated the segregation of subtypes across these clusters: the Basal‐like subtype was primarily concentrated in cluster 1, whereas the LumA, LumB, Her2, Normal, and unknown subtypes, which are clinically distinct from Basal‐like, were predominantly located in cluster 0 (Figure 2j; Table S1, Supporting Information). The consistency of this clustering outcome was observed across the additional 2 molecular subtype identification methods (Figure S4, Supporting Information). Regarding gliomas, our cluster analysis postulated a possible alignment of Lower Grade Glioma (LGG) and Glioblastoma Multiforme (GBM) along a tumorigenic timeline continuum. To investigate this hypothesis, we conducted a pseudotime analysis, which revealed that LGG typically exhibited lower pseudotime values, while GBM presented higher values. This pattern potentially mirrors a disease progression trajectory in gliomas from LGG to GBM (Figure 2k,l). These findings imply that coding lncRNAs have the potential to distinguish between specific subtypes or clinical stages of certain cancers, thereby providing valuable insights into the molecular characterization and classification of various cancers.

### 2.3 Tumor Classification, Typing, and Grading Based on Coding LncRNAs

**Figure 3:**
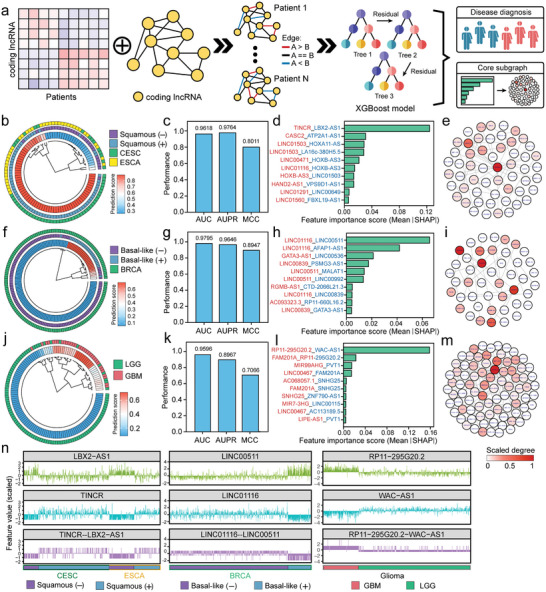
Clinical diagnostics with coding lncRNA graphs. a) The illustration of the graph‐based model for disease diagnosis and core subgraph analysis based on coding lncRNA. b) The circle heatmap showed the scores of squamous cell carcinoma patients from ESCA and CESC in the graph‐based model. c) The bar plot showed the performance of the model for classifying squamous and non‐squamous samples in terms of AUC, AUPR, and MCC. d) The bar plot indicated the importance of each relationship (the edge in the graph) for classification. e) The network showed the subgraph consisting of relationships with an importance score >0. f) The circle heatmap showed the scores of basal‐like and the other patients from BRCA. g) The bar plot showed the performance of the model for typing basal‐like and the other samples in BRCA. h) The bar plot indicated the importance of each relationship for typing. i) The network showed the subgraph consisting of relationships with an importance score >0. j) The circle heatmap showed the scores of LGG and GBM patients. k) The bar plot showed the performance of the model for grading. l) The bar plot indicated the importance of each relationship for grading. m) The network showed the subgraph consisting of relationships with an importance score >0. n) Scaled value of 6 coding lncRNA expression and three pairs of relationships (the edge in the graph).

Given the potential of coding lncRNAs in tumor classification, typing, and grading, we designed a graph‐based model for personalized cancer diagnosis. The model initially constructed a graph for every patient based on the relative expression rank between different coding lncRNAs. This graph was then employed as a feature input of each patient into an XGBoost model for disease diagnosis and subgraph analysis (**Figure** **3**a).

In the task of predicting squamous cell carcinoma patients from ESCA and CESC, the model assigned high scores to squamous and low scores to non‐squamous (Figure 3b; Table S2, Supporting Information). Furthermore, patients from the same cancer type exhibited more similar scores. The model demonstrated impressive performance (AUC: 0.9618, AUPR: 0.9764, and MCC: 0.8011, Figure 3c). Then, the SHAP value analysis identified the relationship (Edge, TINCR – LBX2‐AS1) as the most crucial contributor to disease diagnosis (Figure 3d; Table S2, Supporting Information), with all contributing relationships forming a core subgraph and LINC00467 positioned at the hub center (Figure 3e). Similarly, in typing Basal and non‐Basal patients from BRCA, the model exhibited robust performance (AUC: 0.9795, AUPR: 0.9646, and MCC: 0.8947, Figure 3f,g), with the relationship (Edge, LINC01116 – LINC00511) emerging as the most significant contributor to disease diagnosis, and LINC01116 at the subgraph hub (Figure 3h,i; Table S3, Supporting Information). In grading LGG and GBM patients, the model also displayed high predictive accuracy (Figure 3j,k). The relationship (Edge, RP11‐295G20.2 – WAC‐AS1) was identified as the most critical relationship, with LINC00467 at the center of the subgraph (Figure 3l,m; Table S4, Supporting Information). In addition, these identified coding lncRNAs exhibit distinct expression patterns across a range of cancer subtypes mentioned above, demonstrating a strong association with specific subtypes (Figure S5, Supporting Information).

Furthermore, we analyzed the relative value of the edge in the graph and the absolute value of coding lncRNA expression, according to the most crucial relationship identified in the above three tasks. The results showed that the relative value was more stable across different samples than the absolute value, which is crucial for clinical testing and wide applicability (Figure 3n).

### 2.4 Exploring Spermatogenesis‐Related Coding LncRNAs

**Figure 4:**
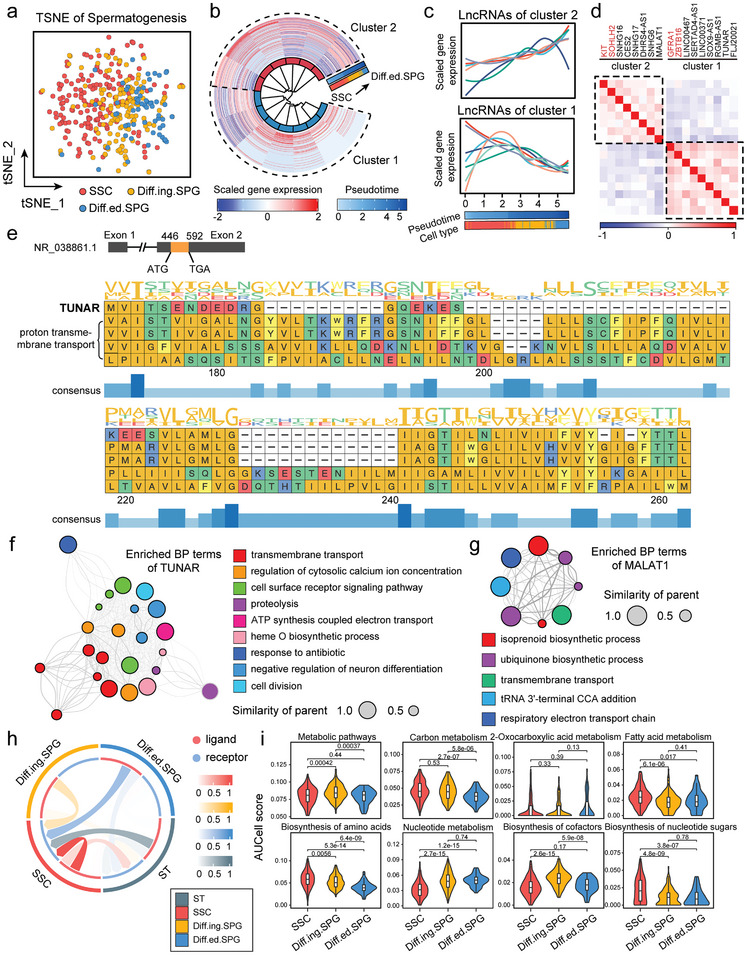
Exploring coding lncRNAs in spermatogenesis. a) The t‐SNE plot visualized cells of spermatogenesis RNA‐seq data for coding lncRNAs. Cells were colored by cell types. b) The circle heatmap represented gene expression during pseudotime of spermatogenesis. c) Two separate clusters (cluster 1 and cluster 2) were used to illustrate lncRNA expression patterns over pseudotime using line charts. d) A heatmap illustrated the correlation between gene expression trends of two lncRNA clusters and markers of spermatogenesis. e) This illustration showed the alignment of TUNAR peptide sequences to the UniProt database. f) A network diagram showcased biological processes primarily associated with TUNAR. g) A network diagram showcased biological processes primarily associated with MALAT1. h) Chord diagrams demonstrated the intensity of intercellular communication during spermatogenesis. i) Violin plots displayed dynamic metabolic pathway enrichment in germ cells throughout spermatogenesis, with p‐values from a two‐sided t‐test.

In addition to documenting associations with various diseases, recent research has also shed light on the role of coding lncRNA in cellular differentiation and development.^[^ ^3^, ^19^ ^]^ Consequently, we attempted to re‐analyze scRNA‐seq data of testicular cells consisting of 354 cells and 134 coding lncRNAs from early spermatogonia, including spermatogonial stem cell (SSC), differentiating spermatogonia (Diff.ing.SPG) and differentiated spermatogonia (Diff.ed.SPG). Dimensionality reduction analysis based on coding lncRNAs indicated a general trend of separation among cells at different spermatogenesis stages (**Figure** **4**a; Figure S6, Supporting Information). To identify coding lncRNAs involved in spermatogenesis, we employed Spearman Correlation analysis and identified 13 coding lncRNAs significantly correlated with pseudotime (Figure 4b). Subsequent hierarchical clustering yielded two groups with divergent expression trends: cluster 1, where expression decreased during SSC differentiation, and cluster 2, which showed an opposite expression trend (Figure 4c). Additionally, we integrated canonical markers of sperm stemness (GFRA1, ZBTB16) and differentiation (KIT, SOHLH2) into our analysis alongside these 13 coding lncRNAs to explore similar expression pattern modules (Figure 4d). The results showed that cluster 1 aligned with genes maintaining stemness (GFRA1, ZBTB16, highlighted in red text), while cluster 2 aligned with genes promoting differentiation (KIT, SOHLH2, highlighted in red text). This correlation further underscores the role of these coding lncRNAs in the developmental process of spermatogenesis.

To further explore the functions of spermatogenesis‐related coding lncRNAs, we specifically focused on TUNAR and MALAT1, from clusters 1 and 2, respectively. Based on the hypothesis that a peptide's function depends on its sequence, we analyzed peptides translated from TUNAR and MALAT1 by conducting BLAST searches in the UniProt database.^[^ ^20^ ^]^ This yielded 250 and 28 similar peptides for TUNAR and MALAT1, respectively (Tables S5 and S6, Supporting Information). For TUNAR, we showcased a portion of these alignment results (Figure 4e). Then, we inferred the functions of these peptides by analyzing the enriched biological process (BP) terms of their similar peptides. The results from the BP functional enrichment analysis suggested that TUNAR peptides might primarily be involved in transmembrane transport and cell surface receptor signaling pathways during spermatogenesis (Figure 4f; Figure S7a, Supporting Information). Furthermore, we analyzed the intercellular communication between SSCs and Sertoli cells (ST), finding that communication intensity progressively diminished during spermatogenesis, paralleling the expression trend of TUNAR (Figure 4h). Meanwhile, peptides similar to MALAT1 were significantly enriched in biosynthetic processes and respiratory electron transport chains, suggesting a potential involvement of MALAT1 in cell metabolism‐related processes (Figure 4g; Figure S7b, Supporting Information). Further single‐cell pathway analysis of 8 metabolic pathways revealed significant dynamic alterations in metabolic processes during spermatogonial development (Figure 4i).

### 2.5 Development of the Platform for Broader Data Analysis

**Figure 5:**
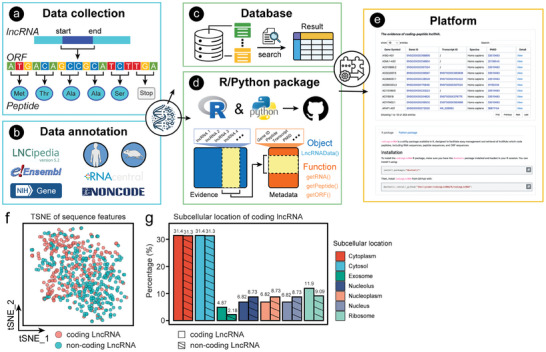
The platform and characteristics of coding lncRNA. a) Annotation of gathered coding lncRNA, including transcript sequences, ORFs, and their relevant peptides. b) Gene and transcript IDs were annotated using databases such as LNCipedia, Ensembl, NCBI, RNAcentral, and NONCODE for coding lncRNAs of human and mouse. c) The database with an efficient search functionality and secured with HTTPS protocol. d) The detailed structure of object and function in R and Python packages. e) The image of the database and package in codLncWeb. f) The t‐SNE plot of coding lncRNA and non‐coding lncRNA using 49 features. g) The percentage of 7 subcellular localizations of coding lncRNA and non‐coding lncRNA, respectively.

To advance the widespread application and analysis of coding lncRNAs, we have established codLncWeb (https://cellknowledge.com.cn/codingLncRNA/), an online platform dedicated to storing, browsing, and exploring coding lncRNAs. codLncWeb incorporated the knowledge of 353 experimentally validated coding lncRNAs, complete with detailed annotations such as gene and transcript IDs, ORFs, peptide sequences, and associated reference lists (**Figure** **5**a,b). codLncWeb delivered an exceptional user experience through its asynchronous database architecture, ensuring rapid data retrieval while concurrently upholding stringent user privacy via the HTTPS protocol (Figure 5c). Moreover, codLncWeb also embedded the packages designed for compatibility with the R and Python programming environments (Figure 5d). The core structure of packages, LncRNAData, enabled the extraction of necessary information using functions like getRNA, getPeptide, and getORF. Finally, we deployed the above tools to an easily accessible website, complete with usage instructions (Figure 5e).

Based on the codLncDB, we analyzed the characteristics and subcellular location of coding lncRNAs, revealing differences between coding and non‐coding lncRNAs. Specifically, we observed variances of sequence characteristics between the two lncRNA classes, but not obvious (Figure 5f). Furthermore, we predicted the subcellular location of these lncRNAs and found that they have a greater tendency to localize with ribosomes than non‐coding lncRNAs (Figure 5g).

### 2.6 Continuously Collecting Coding LncRNA with a Knowledge‐Mining Tool

**Figure 6:**
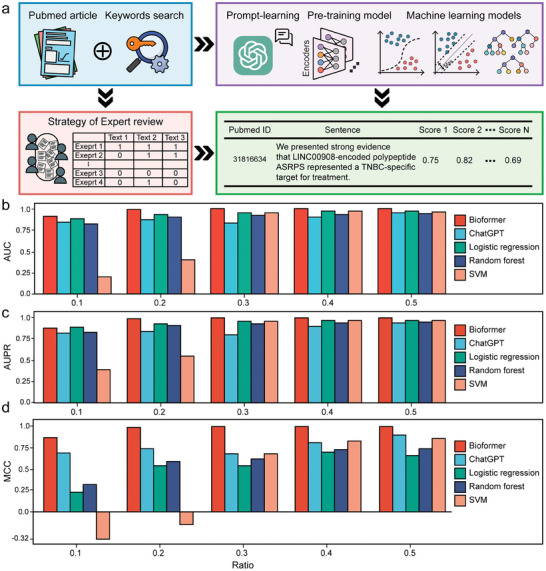
NLP models for timely update of coding lncRNA. a) After conducting a keyword search on PubMed, utilized a combination of expert reviews and NLP models to score sentences. b–d) Evaluation of various NLP models, including traditional machine learning models, a pre‐training model, and a ChatGPT 4.0‐based prompt‐learning model, with bar charts of AUC, AUPR, and MCC. The *x*‐axis represents the ratio of the dataset used for model training in few‐shot evaluation strategies.

The field of research on coding lncRNAs is rapidly expanding, with new coding lncRNAs being continually identified.^[^ ^7^, ^21^ ^]^ However, relying solely on manual mining is time‐consuming and inefficient. To address this challenge and facilitate the timely update, we have implemented a novel text mining approach, codLncNLP. This method synergizes expert review with state‐of‐the‐art NLP models to streamline the data‐updating process (**Figure** **6**a). Our integration included traditional machine learning methods such as logistic regression, support vector machines (SVM), and random forest, along with the pre‐training model, Bioformer,^[^ ^15^ ^]^ and the prompt‐learning model based on ChatGPT 4.0.^[^ ^16^ ^]^ Given the limited scope of the benchmark dataset (160 sentences, see detail in the Experimental Section), we adopted a few‐shot evaluation strategy. This strategy involved using varying percentages of the dataset as the training set, ranging from 10% to 50%, the remaining as the testing set. Overall, the pre‐training model, Bioformer, consistently surpassed other models in terms of AUC, AUPR, and MCC across different training set sizes (Figure 6b–d; Table S7, Supporting Information). In addition, the logistic regression showed higher AUC and AUPR values but lagged in MCC. The prompt‐learning model displayed the reverse pattern, with lower AUC and AUPR but higher MCC.

## 3 Discussion

Recent studies have extensively demonstrated that under certain conditions, some lncRNAs are capable of producing peptides, playing varied biological roles in both disease and physiological processes.^[^ ^22^ ^]^ However, current research often concentrates on the identification and functional analysis of translational events in individual lncRNAs, leading to a noticeable gap in the systematic exploration and analysis of coding lncRNAs. Therefore, this study introduces codLncScape, a well‐functioning and self‐enriching framework designed to aid in the research of coding lncRNAs. codLncScape begins by constructing a manually curated dataset of coding lncRNAs, followed by a systematic investigation into their expression and function in both pathological and physiological contexts. This represents a preliminary compilation and summary of currently identified coding lncRNAs, offering a solid foundation for the future identification and functional analysis of new coding lncRNAs. Moreover, codLncScape developed a platform, releasing the database and packages for use in various programming environments, along with the establishment of NLP tools to assist in the timely integration and updating of knowledge. The ultimate goal of codLncScape is to build a rich and fully functional portal where researchers can freely and conveniently access, learn, and exchange the latest advancements and knowledge on coding lncRNAs, thereby further advancing the research in this field.

Our study in the pan‐cancer genome revealed that coding lncRNAs not only demonstrated good classification capabilities for different tumor types and cell origins but also distinguished certain tumor subtypes and clinical stages. Furthermore, the personalized diagnosis model developed based on coding lncRNAs further confirmed the predictive effectiveness of coding lncRNAs in tumor subtyping and staging. These results suggest that coding lncRNAs are closely associated with the development of various cancers and hold the potential for clinical early warning and diagnosis. Similarly, systematic analysis of coding lncRNAs in spermatogenesis‐related scRNA‐seq data identified several coding lncRNAs highly related to the spermatogenic process. Further analysis exploring the functions of peptides expressed by these coding lncRNAs indicated that these translation products might be involved in intercellular communication and metabolic processes during spermatogenesis. These findings suggest that coding lncRNAs, through their translated peptides, may play a role in regulating cellular differentiation and development, meriting further investigation.

Additionally, this study systematically collated and annotated currently identified coding lncRNAs and developed an integrated data resource platform. On this platform, a series of data management and analysis toolkits were developed, to fill the gap among different programming environments and enhance interdisciplinary communication. Our findings also showed that coding lncRNAs are preferred for coding over other RNA sequences, in terms of specific intrinsic RNA sequence attributes and subcellular localizations. Feature extraction and constructing models specifically for coding lncRNAs may pose a significant challenge in future research endeavors.^[^ ^23^ ^]^ Furthermore, this study also integrated machine learning, pre‐training models, and prompt‐learning methods to develop a knowledge‐mining tool for coding lncRNAs, self‐enriching the content of codLncDB timely and efficiently.

This study still has some limitations. First, the collected coding lncRNAs, validated by low‐throughput experiments, are limited in number, and inevitably carry certain research biases, such as many documented coding lncRNAs are related to tumorigenesis. Additionally, our current research predominantly focuses on the expression of coding lncRNAs in various physiological and pathological states, yet it lacks a comprehensive analysis of the translational dynamics and specific functions of the peptides these lncRNAs encode. In the future, we plan to further collect coding lncRNA data, expand our dataset, and enhance the prediction and exploration of the functions of their translated peptides.

In summary, this study not only compiled a manually curated knowledge base of coding lncRNAs but also systematically explored their roles in disease and physiology. It developed and integrated a series of tools and workflows for data collection, management, and analysis around coding lncRNAs. Our aim is to construct a user‐friendly and content‐rich ecosystem focused on this research hotspot, progressively providing data foundations and technical support for the mechanistic and functional elucidation of coding lncRNAs.

## 4 Experimental Section

### Establishment of codLncDB

The coding lncRNA knowledge base, codLncDB, which the study developed, was meticulously curated from the literature and five distinct databases. For curation, the following keyword combinations were used to filter publications in PubMed (mainly from 2018 to 2023): (encoded [Title/Abstract]) AND (lncRNA [Title/Abstract]), (peptide [Title/Abstract]) AND (lncRNA [Title/Abstract]), (translation [Title/Abstract]) AND (lncRNA [Title/Abstract]). Then, all retrieved publications were preliminarily reviewed by expert curators to filter out false‐positive papers. Only the experimentally supported coding lncRNAs (the peptide was detected by low throughput experiments) were collected, resulting in 293 entries. Additionally, another 60 coding lncRNA entries were integrated from five databases, including ncEP,^[^ ^10^ ^]^ FuncPEP,^[^ ^11^ ^]^ EVLncRNAs 2.0,^[^ ^12^ ^]^ cncRNAdb,^[^ ^8a^ ^]^ and LncPep.^[^ ^13^ ^]^ At last, 353 entries of coding lncRNA entries (337 human, 16 mouse) were documented, involving 329 lncRNAs (Figure S1, Supporting Information).

To unify the coding lncRNA from multiple sources in authoritative reference databases, we mapped all the lncRNAs to five databases (Ensembl,^[^ ^24^ ^]^ NCBI Gene,^[^ ^25^ ^]^ RNAcentral,^[^ ^26^ ^]^ NONCODE,^[^ ^27^ ^]^ and LNCipedia^[^ ^28^ ^]^) to annotate coding lncRNA and their corresponding transcripts. In the annotation process, the IDs used in the original literature were prioritized to ensure accuracy and consistency throughout the annotation process. Additionally, the codLncDB database was compared against existing resources,^[^ ^8^, ^10^, ^11^, ^12^, ^13^, ^29^ ^]^ to underscore its distinctive advantages. This analysis delved into three critical dimensions: the source of evidence, involved species, and data categories. The outcomes of this comparison are meticulously detailed in Figure S8 (Supporting Information).

### Pan‐Cancer RNA Sequencing Data Collection

The pan‐cancer transcriptome data of TCGA was downloaded from the pancancer_xena platform,^[^ ^30^ ^]^ which involves 33 types of cancer, sourced from the IlluminaHiSeq platform. All data analyses in this study were based on TPM values. The clinical metadata, including the cell origin and cancer subtype, were also compiled for the entire cohort.

### Pan‐cancer RNA Sequencing Data Processing

The 329 coding lncRNAs were intersected with genes detected in the pan‐cancer transcriptome. This process required the inclusion of lncRNAs detected in at least three samples for further analysis. Subsequently, a cohort of 140 coding lncRNAs exhibiting this overlap was obtained, culminating in a profile including 9,784 samples across 33 cancer types.

To further analyze pan‐cancer data, the cluster analysis strategy was used in Seurat4 package (v4.2.0).^[^ ^31^ ^]^ The log‐transformed (log1p) TPM expression data was assigned to the data slot of the Seurat object. To extract features of the coding lncRNA profile in pan‐cancer, the Seurat functions ScaleData and RunPCA were employed. Given the insufficiency of original features, the parameter ‘features’ was set to 140 in RunPCA. Then, the Seurat function FindNeighbors (dims = 1:20) was used to construct a shared nearest neighbor graph and the Seurat function FindClusters (resolution = 0.5) to identify clusters. PCs 1–20 were selected to perform t‐SNE analysis with the Seurat function RunTSNE (perplexity = 30).

### Tumor Clustering and Purity

To explore the relationship between different cancer types and clusters, TCS and TPS were calculated for all clusters. First, for the *i*‐th cancer type, the TCS is calculated as follows:

(1) $$TCS_{i,j}=\left(T_{i,j}\;/T_{i}\right)\times 100\%$$

(2) $$TCS_{i}=\;max\left(TCS_{i,j}\;|\;j\in \left\{1,2,,,m\right\}\right)$$

where *T_i_* is the total number of samples for the *i*‐th cancer type, and *T*_*i*,*j*_ is the number of samples of the *i*‐th cancer type within the *j*‐th cluster. *TCS*_*i*,*j*_ represents the percentage of the *i*‐th cancer type sample assigned to the *j*‐th cluster and *TCS_i_* represents the max percentage of the *i*‐th cancer type across all m clusters; a higher *TCS_i_* value indicates stronger clustering ability. TCS represents the clustering ability of coding lncRNAs for a specific cancer type; a higher TCS value indicates a stronger clustering ability.

Simultaneously, for the *j*‐th cluster, the TPS is computed as follows:

(3) $$TPS_{i,j}=\left(T_{i,j}\;/C_{j}\right)\times 100\%$$

(4) $$TPS_{j}=\;max\left(TPS_{i,j}\;|\;i\in \left\{1,2,,,n\right\}\right)$$

where *C_j_* is the number of samples contained within the *j*‐th cluster, and *T*_*i*,*j*_ is the number of samples of the *i*‐th cancer type within the *j*‐th cluster. *TPS*_*i*,*j*_ represents the percentage of the *i*‐th cancer type sample in the *j*‐th cluster and *TPS_j_* represents the max percentage of the *j*‐th cluster across all n cancer types; a higher *TPS_j_* value indicates higher tumor purity. If the TPS for any given cluster exceeds 50%, it suggests that the cluster has a dominant cancer type and can be termed a cancer‐associated cluster. If the TPS is below 50%, the cluster is considered a hyper‐cluster composed of a mix of various cancer types.

### Enrichment Score of Squamous Cell Samples across Clusters

For analyzing the enrichment of squamous cell samples across different clusters, we used the *R*_*O*/*E*_ (Observed value divided by expected value) method to evaluate the enrichment between clusters and tumor types. The expected value, *E*_*i*,*j*_, represents the number of the *i*‐th cancer type squamous cell samples expected to appear in the *j*‐th cluster under random conditions. In contrast, the observed value, *O*_*i*,*j*_, represents the number of the *i*‐th cancer type squamous cell samples in *j*‐th cluster. The $R_{O/E}^{i,j}$ is calculated as follows:

(5) $$E_{i,j}=\left(\frac{T_{j}}{T}\right)\;\times S_{i}$$

(6) $$R_{O/E}^{i,j}=\frac{O_{i,j}}{E_{i,j}}$$

where *T_j_* represents the total number of samples in the *j*‐th cluster; *T* represents the total number of samples across different clusters; *S_i_* represents the number of squamous cell samples for the *i*‐th cancer type; $R_{O/E}^{i,j}$ represents the enrichment score of squamous cell samples between the *i*‐th cancer type and the *j*‐th cluster. If the enrichment score is >1, it indicates an enrichment relationship between a cluster and a tumor type.

### Molecular Subtype Analysis of Breast Cancer

TCGA's annotated breast cancer molecular subtype clinical data (PAM50_mRNA_nature2012 and PAM50Call_RNAseq) was used to analyze 518 and 843 samples, post‐exclusion of unknown data. Basal subtype samples were designated class 1 (positive samples), and non‐basal subtype samples were class 0 (negative samples). The unsupervised clustering outcomes (clusters 0 and 1) served as predictive variables, facilitating the computation of accuracy, F1 score, Matthew's correlation coefficient (MCC), precision, and recall under both molecular subtypes. Furthermore, the basal subtype signature, M8124 in MSigDB,^[^ ^32^ ^]^ was employed using single‐sample enrichment analysis methods to calculate each sample's basal score, correlating higher scores with basal subtype similarity. Then, we computed correlation coefficients and statistical significance of the two‐sided Spearman test.

### Graph‐Based XGBoost Diagnosis Model

To construct a cancer diagnostic model based on coding lncRNAs, the study developed a machine‐learning model, codLncDisease, which used a graph composed of coding lncRNAs as the feature input for each patient into the XGBoost model. The specific workflow is as follows:

For a coding‐lncRNA graph G, the study defines:

(7) $$G\;=\left(V,E,W\right)$$

(8) $$V\;=\left\{v_{1},v_{2},\text{…},\left.v_{n}\right\}\right.$$

(9) $$E\;=\left\{e_{ij}|v_{i},v_{j}\in V,i<j\right\}$$

(10) $$W:\;E\times I\to \left\{-1,0,1\right\}$$

The weight function $W_{\left(e_{ij},k\right)}$ is set according to the following rules:

(11) $$W_{\left(e_{ij},k\right)}=\left\{\begin{array}{c} 1,\;if\;x_{i,k}-x_{j,k}>1\\ -1,\;if\;x_{i,k}-x_{j,k}<-1\\ 0,\;otherwise \end{array}\right.$$

where *G* is the graph used as input for the codLncDisease model, *V* represents the set of nodes encompassing all the coding‐lncRNAs {*v*_1_,*v*_2_,…, *v_n_*}, *E* represents the set of edges between nodes, and *e_ij_* indicates the edge between nodes *v_i_* and *v_j_*, with the count being $C_{n}^{2}$. The weight function *W* assigns a weight to each edge in E for each patient. *x*_*i*,*k*_ and *x*_*j*,*k*_ are the expression values of lncRNAs *v_i_* and *v_j_*, within patient *k*, assigning different weights to the edge based on their expression difference. By employing this strategy, which utilizes the relative ranking information between different coding lncRNAs to construct a tumor diagnostic model, the impact of experimental techniques on the model's performance can be significantly reduced, effectively enhancing the model's robustness. Ultimately, we feed the graph into the XGBoost model for accurate prediction of tumor types:

(12) $$XGBoost\;\left(G\right)=\;XGBoost\left(V,E,W\right)$$

The codlncDisease model allocates scores ranging from 0 to 1 for every sample. The performance metrics for the models include AUC (Area Under Curve), AUPR (Area Under Precision‐Recall Curve), and MCC (Matthews Correlation Coefficient), all implemented via the sklearn.metrics module in the scikit‐learn package (v1.1.1).

### Identified Core Subgraph of Coding‐LncRNA Graph

SHAP value was a game‐theoretic approach to explaining the machine learning model. The SHAP module (v0.43.0) was employed to unearth the core subgraph within the coding‐lncRNA graph. The SHAP function explainer was used to compute the model's feature importance score (Mean|SHAP), indicating the average contribution of each feature to the model's prediction, with larger values representing greater importance. Then, all features were selected with SHAP values >0 to create a core subgraph.

### Spermatogenesis scRNA‐seq Data Collection and Processing

The scRNA‐seq data of human spermatogenesis by modified smart‐seq2 technology were collected from the NCBI GEO database (GSE106487).^[^ ^33^ ^]^ The study obtained 354 cells across stages of SSC, Diff.ing.SPG, Diff.ed.SPG and the expression levels were normalized by log2[TPM/10+1] (transcripts per million, TPM). Then an intersection analysis of 329 coding lncRNAs was pursued with the genes detected within the spermatogenesis transcriptome, requiring each lncRNA to be expressed in at least three cells for further analysis. Subsequently, a cohort of 134 coding lncRNAs exhibiting this overlap was obtained, culminating in a coding lncRNA profile that included 354 cells across three stages.

The scRNA‐seq data were processed by Seurat4 (v4.2.0). The log‐transformed (log1p) TPM expression data was assigned to the data slot of the Seurat object. To extract the primary features of the coding lncRNA profile, the Seurat functions ScaleData and RunPCA were employed. Given the insufficiency of original features, the parameter ‘features’ was set to 134 in RunPCA. Then PCs 1–20 were selected for t‐SNE analysis with the Seurat function RunTSNE (perplexity = 70). Cell identities were obtained from the original paper.

### Correlation Analysis of Coding LncRNA

To identify the coding lncRNA associated with pseudotime, the stats package (v4.2.1) was used to calculate Spearman correlation coefficients between gene expression and pseudotime. The study screened for pseudotime‐associated coding lncRNA by setting the threshold (|r| > 0.2, p < 0.05). Next, the canonical marker genes of SSC stemness, GFRA1, and ZBTB16 were combined with the canonical marker genes of spermatogonia differentiation, KIT and SOHLH2, as well as the pseudotime‐associated coding lncRNA. Spearman correlation coefficients and performed unsupervised clustering were calculated using the corrplot package (v0.92).

### BLAST Analysis of Peptides Encoded by Coding LncRNAs

To search for peptide sequences analogous to those translated from TUNAR (ORF 446–592) and MALAT1 (ORF 3086–3229), the “blastp” tool and selected “UniProtKB reference proteomes” along with “Swiss‐Prot” were employed as the reference databases (including both reference proteomes and proteins that were reviewed).^[^ ^34^ ^]^ The E‐value threshold was set at 10, consistent with the default settings.

### Functional Enrichment of LncRNA‐coding Peptide

To elucidate the potential biological functions of the lncRNA‐coding peptides, the Gene Ontology biological process term (BP term)^[^ ^35^ ^]^ of similar peptides was used as possible functions for the peptide of TUNAR and MALAT1. To make the functional annotation easier to understand and streamlined, the rrvgo package (v1.8.0)^[^ ^36^ ^]^ was used to calculate the similarity between different BP terms. Subsequently, the study clustered the BP terms and nominated the most prominent BP term within each cluster as the representative (Parent term).

### Analysis of Cell Communication

For the analysis of cellular communication, ST cells were incorporated, which were integral in establishing the early microenvironment for SSC in the testicular niche. Utilizing the CellCall package (v1.0.0),^[^ ^37^ ^]^ the complete expression profile across the various stages of spermatogenesis was analyzed, including SSC, Diff.ing.SPG, Diff.ed.SPG, and ST cells. Subsequently, the TransCommuProfile and ViewInterCircos functions were applied within the CellCall package to deduce potential ligand‐receptor interactions among the cell types and to evaluate the comprehensive communication intensity.

### Analysis of Metabolic Pathway Activity

To determine the activation of each sub‐metabolic pathway in every cell, the AUCell package (v1.18.1)^[^ ^38^ ^]^ was employed to calculate metabolic pathway activity and used a two‐sided t‐test in the stats package (v4.2.1) to assess the statistical significance between the two groups. A total of 8 metabolism pathways from the Kyoto Encyclopedia of Genes and Genomes (KEGG) were collected for the activity analysis,^[^ ^39^ ^]^ including Metabolic pathways (hsa01100), Carbon metabolism (hsa01200), 2‐Oxocarboxylic acid metabolism (hsa01210), Fatty acid metabolism (hsa01212), Biosynthesis of amino acids (hsa01230), Nucleotide metabolism (hsa01232), Biosynthesis of nucleotide sugars (hsa01250), and Biosynthesis of cofactors (hsa01240) (Table S8, Supporting Information).

### Development of codLncWeb

The architecture of the platform was built upon the HTTPS protocol, ensuring secure access for users. Its search functionality was powered by the DataTables framework, leveraging JavaScript for asynchronous data querying, thus optimizing both search efficiency and user‐friendliness. The platform embedded an innovative resource manager codLncPackage tailored for compatibility with the R and Python programming environments. Its core structure, LncRNAData, encapsulates comprehensive information on transcripts, peptides, and ORFs, allowing users to effortlessly extract necessary information using methods like getRNA, getPeptide, and getORF. To support and guide users, the study provided extensive documentation and tutorials, available on the official server, ensuring that the platform was not only functional but also user‐centric and accessible.

### Sequence Features and Subcellular Localization

For the analysis of lncRNA sequence features and subcellular localization, the data was downloaded from GENCODE V44, selectively removing lncRNAs listed in codLncDB. After excluding 282 human lncRNAs identified in codLncDB, another 282 lncRNAs were randomly selected as non‐coding samples for analysis. Forty‐nine features of lncRNAs were analyzed as previous research,^[^ ^40^ ^]^ categorizing them into sequence intrinsic properties, physicochemical characteristics, and secondary structures. For subcellular localization predictions, the iLoc‐lncRNA tool was utilized,^[^ ^41^ ^]^ covering seven locations: cytoplasm, cytosol, exosome, nucleolus, nucleoplasm, nucleus, and ribosome. This enabled a comparative analysis of the localization tendencies between coding and non‐coding lncRNAs.

### Development of codLncNLP

A corpus was constructed for the text mining of coding lncRNAs. Initially, 80 positive sentences containing confirmed information about coding lncRNAs were curated. These sentences were manually selected from 77 research papers related to coding lncRNAs. In parallel, 80 negative sentences were compiled, which do not contain information about coding lncRNAs, to serve as negative samples. These were randomly chosen by manually selected from publications found on PubMed using the keyword “lncRNA”. Subsequently, the nltk package was employed for text preprocessing, which included removing special characters, deleting non‐linguistic content, tokenization, and lemmatization. This process resulted in a balanced text corpus comprising 160 sentences (80 positives vs. 80 negatives), which is available in Table S9 (Supporting Information).

codLncNLP integrated 5 NLP models, including three machine‐learning methods (logistic regression, SVM, and random forest), a pre‐training model (Bioformer),^[^ ^15^ ^]^ and a prompt‐learning model (ChatGPT4.0). The machine learning models are implemented in the scikit‐learn package (v1.1.1). For the pre‐training model, we fine‐tuned the bioformers (bioformer‐8L) with our corpus, utilizing the simpletransformers package (v0.64.3). Moreover, a prompt‐learning model was developed based on the ChatGPT‐4.0 architecture,^[^ ^16^ ^]^ with detailed prompts available in Table S10 (Supporting Information). The performance of different models was assessed using the AUC, AUPR, and MCC metrics in sklearn.metrics module.

### Statistical Analysis

All statistical analyses were conducted using R version 4.2.1 (https://www.r‐project.org/). Hierarchical clustering analysis for heatmaps was performed using the “euclidean” and “complete” methods, as implemented in the R package pheatmap (v1.0.12). Pseudotime analysis is performed by the monocle package (v2.24.1).^[^ ^42^ ^]^ Spearman's rank correlation coefficient was employed to measure the relationship between two variables, with the corresponding significance assessed using a two‐sided hypothesis test (|r| > 0.2, p < 0.05), as implemented in the R package corrplot (v0.92). The significant analysis of metabolic pathway activation scores was performed using the t‐test, as implemented in the R package stats (v4.2.1). All p‐values were two‐sided, with p < 0.05 indicating a statistically significant difference.

### Data Availability

The dataset of coding lncRNAs used in this study is freely available at https://cellknowledge.com.cn/codingLncRNA/. The pan‐cancer data used in this study is available at the UCSC Xena Database (https://xenabrowser.net/datapages/), and scRNA‐seq data of spermatogenesis is available at the NCBI Gene Expression Omnibus (GEO) database (GSE106487).^[^ ^33^ ^]^

### Code Availability

The codLncWeb is freely accessible at https://cellknowledge.com.cn/codingLncRNA. All source code used in this paper is also deposited at https://github.com/ShellyCoder/codingLncRNA.

## Conflict of Interest

The authors declare no conflict of interest.

## Author Contributions

Y.Z., H.L., and X.Y. conceived this project. T.L., H.Q., and Z.W. designed and performed the experiments. X.Y. helped with data interpretation and visualization. Y.Z. and T.L. wrote the manuscript. Z.W., X.P., and Y.Y. helped with manuscript reviewing. Y.Z. and T.S. supervised this project.

## Supporting information

Supporting Information

Supporting Information

Supporting Information

Supporting Information

Supporting Information

Supporting Information

Supporting Information

Supporting Information

Supporting Information

Supporting Information

Supporting Information

## Data Availability

The data that support the findings of this study are available in the supplementary material of this article.
