# Supplementary material for: CodLncScape Provides a Self‐Enriching Framework for the Systematic Collection and Exploration of Coding LncRNAs
Source: Adv Sci (Weinh). 2024 Apr 11;11(22):2400009. doi: 10.1002/advs.202400009 (PMC11165466; doi:10.1002/advs.202400009)
Supplement: Supplementary file 1 — Supporting Information [file ADVS-11-2400009-s011.pdf]

ADVANCED SCIENCE
Open Access

Supporting Information

for *Adv. Sci.*, DOI 10.1002/advs.202400009

CodLncScape Provides a Self-Enriching Framework for the Systematic Collection and Exploration of Coding LncRNAs

Tianyuan Liu, Huiyuan Qiao, Zixu Wang, Xinyan Yang, Xianrun Pan, Yu Yang, Xiucai Ye, Tetsuya Sakurai, Hao Lin* and Yang Zhang**

Supporting Information

CodLncScape Provides a Self-Enriching Framework for the Systematic Collection and Exploration of Coding LncRNAs

Tianyuan Liu, *Huiyuan Qiao*, *Zixu Wang*, *Xinyan Yang*, *Xianrun Pan*, *Yu Yang*, *Xiucai Ye**, *Tetsuya Sakurai*, *Hao Lin**, *Yang Zhang**

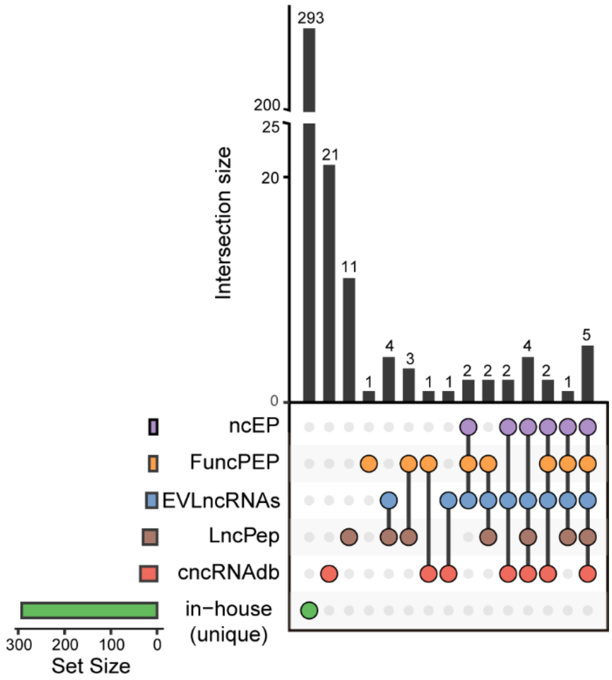

Figure S1. The intersection of unique in-house evidence for coding lncRNA and that from other databases.

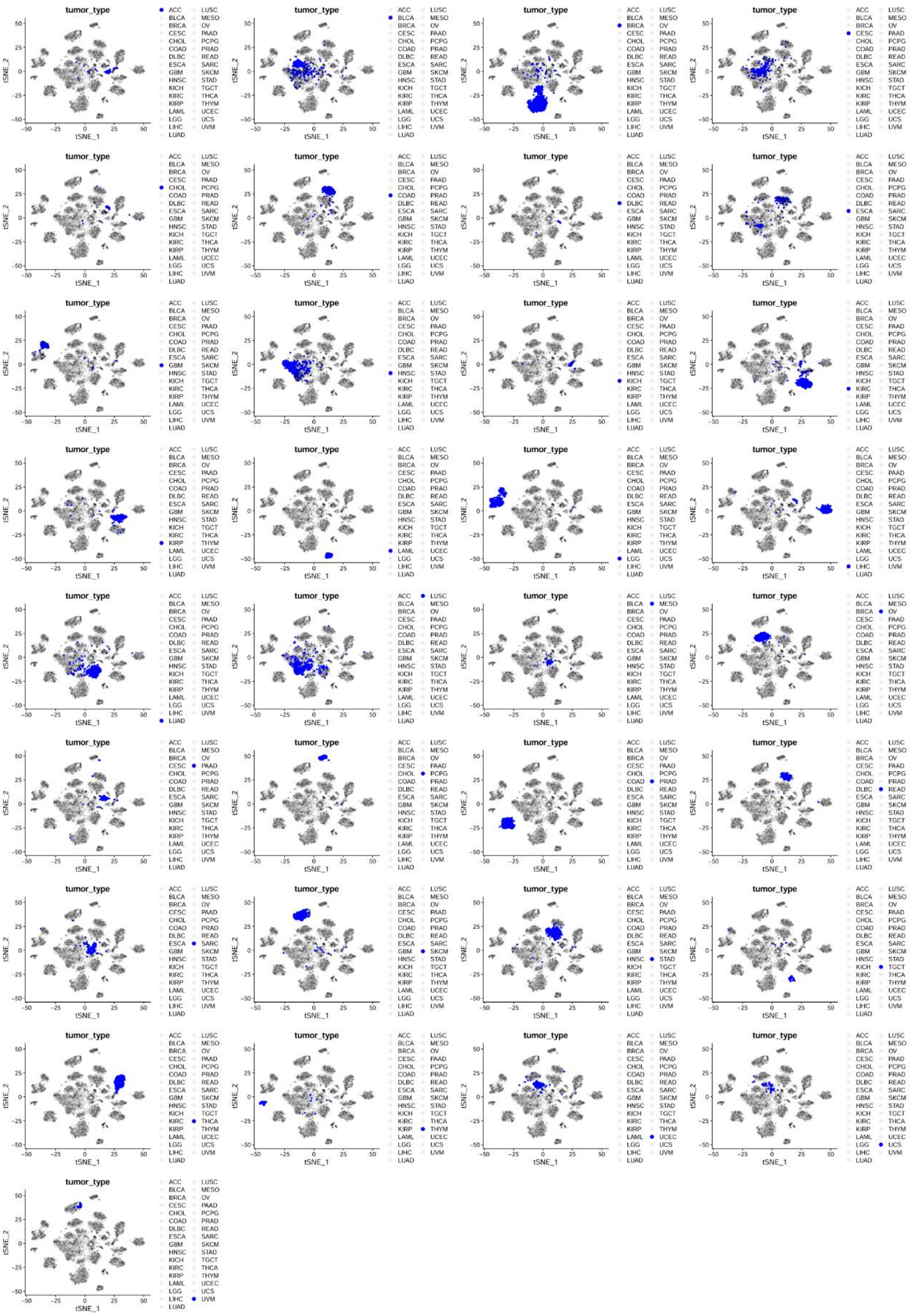

Figure S2. t-SNE visualization of 33 cancer types in the pan-cancer context.

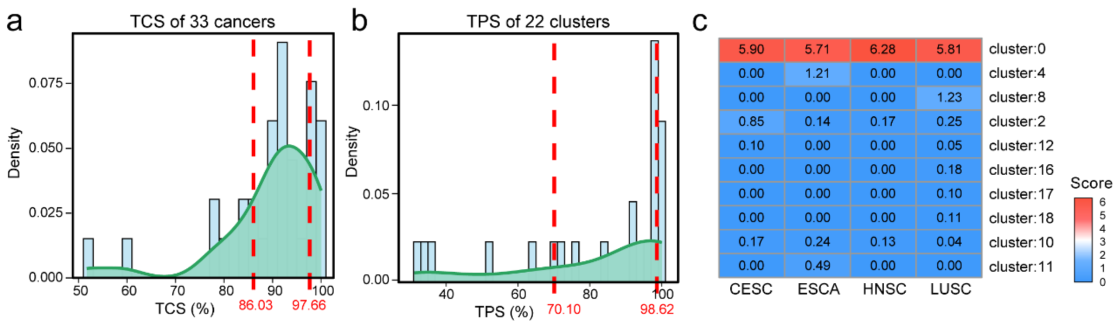

Figure S3. The threshold and preference of clusters. (a) Distribution of the TCS for 33 cancers, with 25th and 75th percentiles: 86.03% and 97.66%, respectively (indicated by red lines). (b) Distribution of the TPS, with 25th and 75th percentiles: 70.10% and 98.62%, respectively (indicated by red lines). (c) Cluster preference of each squamous cell carcinoma sample estimated by Ro/e (ratio of observed and expected samples). The heatmap displays the number of Ro/e values and is colored by Ro/e values.

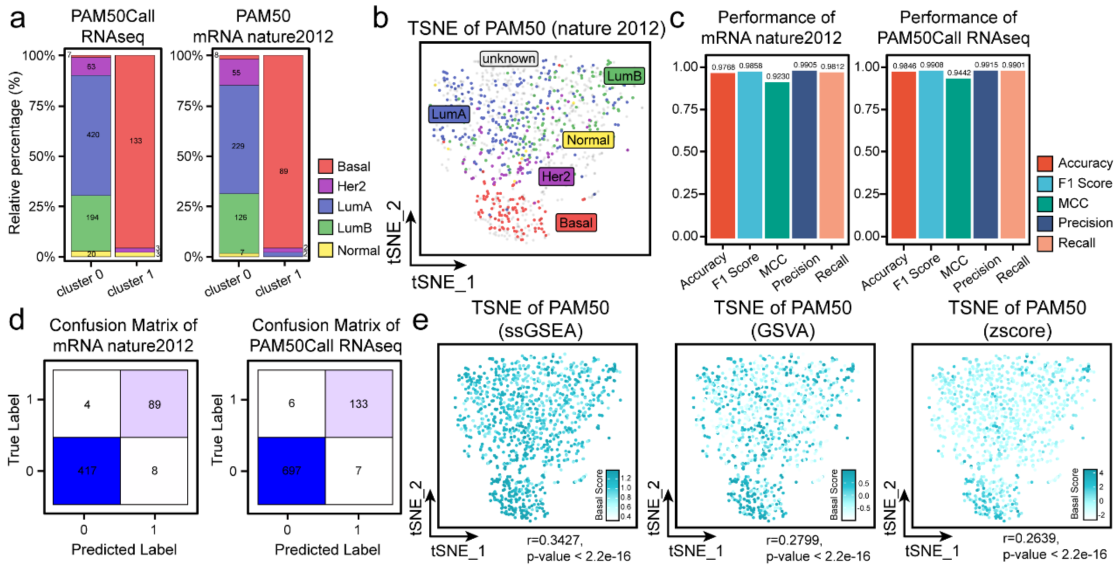

Figure S4. Analysis of unsupervised clustering with breast cancer molecular subtypes. (a) Statistics of breast cancer molecular subtypes from 2 sources: PAM50Call_RNAseq and PAM50_mRNA_nature2012. (b) TSNE plot of breast cancer samples with molecular subtype: PAM50_mRNA_nature2012. (c) The performance and (d) confusion matrix of unsupervised clustering in distinguishing basal and non-basal subtype samples. (e) TSNE plot of breast cancer samples with basal scores, using ssGSEA, GSVA, and zscore based on MSigDB signature. Spearman correlation

coefficients and p-value with the two-sided test.

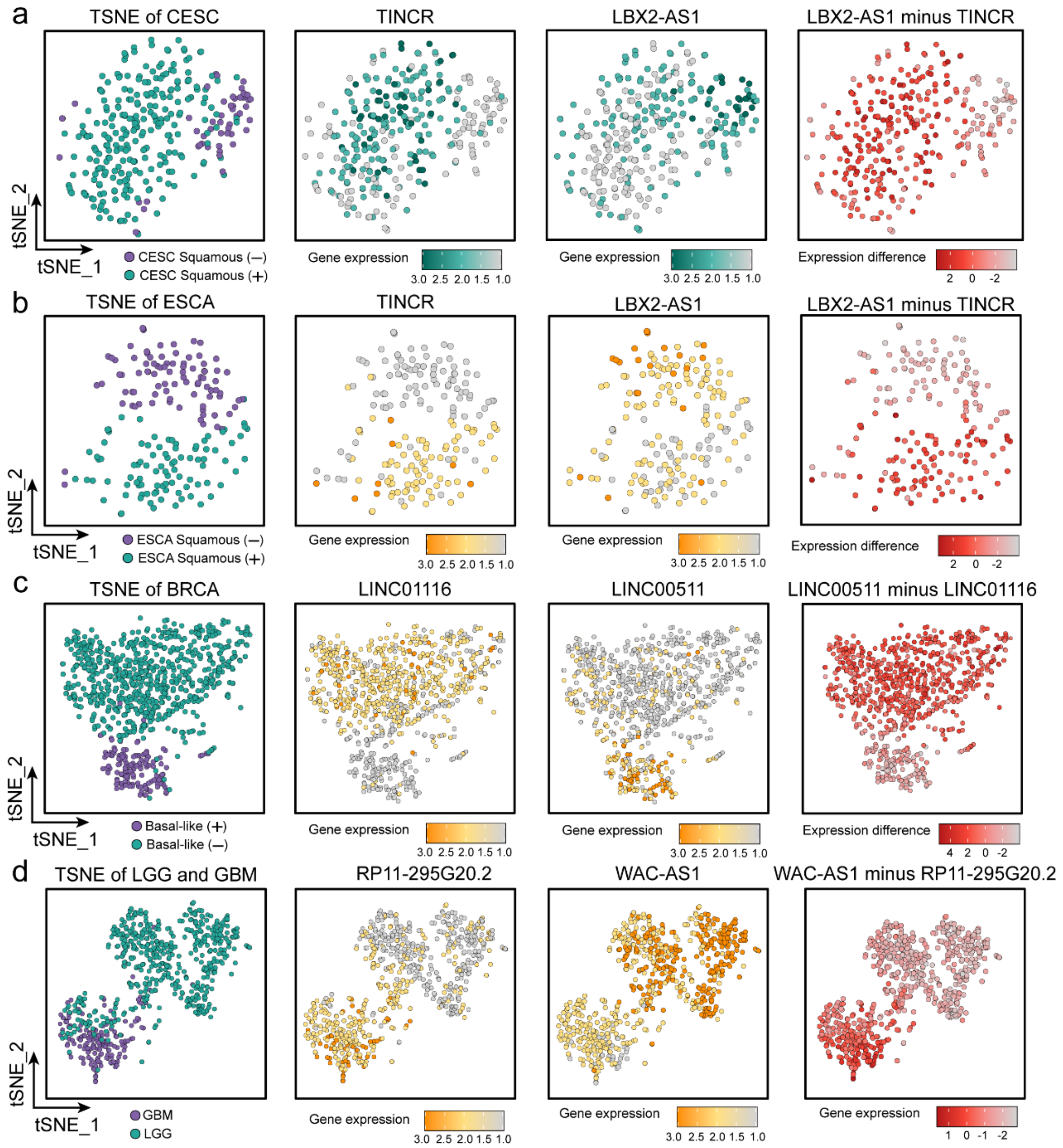

Figure S5. Expression patterns of coding lncRNAs across different cancer subtypes. (a) CESC: The first t-SNE plot showed tumor subtype classification, the second and third t-SNE plots displayed the expression levels of TINCR and LBX2-AS1, respectively, and the fourth t-SNE plot illustrated the expression difference between TINCR and LBX2-AS1. (b) ESCA: The first t-SNE plot showed tumor subtype classification, the second and third t-SNE plots displayed the expression levels of TINCR and LBX2-AS1, respectively, and the fourth t-SNE plot illustrated the expression difference between TINCR and LBX2-AS1. (c) BRCA: The first t-SNE plot

showed tumor subtype classification, the second and third t-SNE plots displayed the expression levels of LINCO1116 and LINC00511, respectively, and the fourth t-SNE plot illustrated the expression difference between LINCO1116 and LINC00511. (d) LGG to GBM: The first t-SNE plot showed tumor subtype classification, the second and third t-SNE plots displayed the expression levels of RP11-295G20.2 and WAC-AS1, respectively, and the fourth t-SNE plot illustrated the expression difference between RP11-295G20.2 and WAC-AS1.

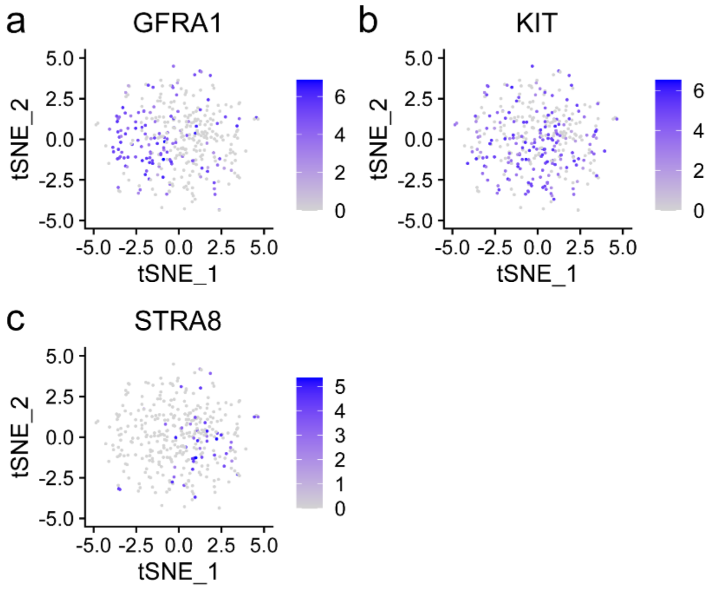

Figure S6. The t-SNE plot of marker genes in spermatogenesis. Cells were colored by gene expression of marker genes in spermatogenesis.

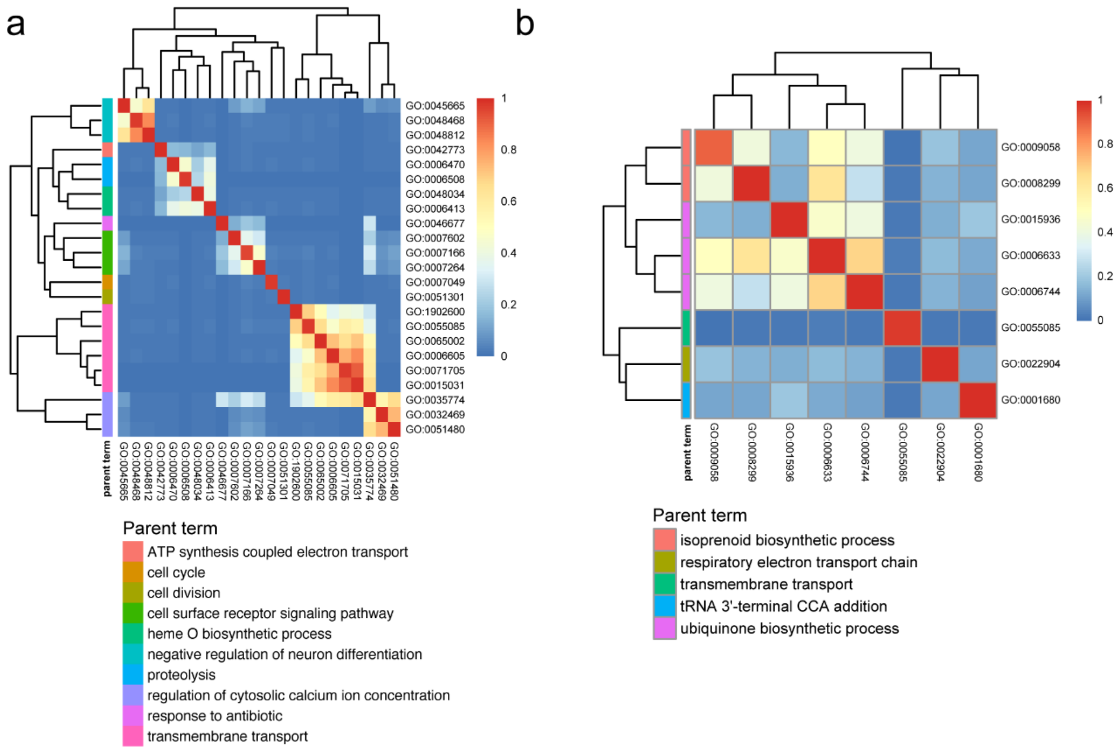

Figure S7. **BP terms of TUNAR and MALAT1.** The heatmaps of the BP terms associated with TUNAR (a) and MALAT1 (b).

Database	Evidence		Sequence		Species		Publication
	Low throughput	High throughput	RNA	Peptide	Human	Others	Year
codLncDB	✓	✗	✓	✓	✓	✓	/
ncEP	✓	✗	✗	✓	✓	✓	2020
FuncPEP	✓	✓	✗	✓	✓	✓	2020
EVLncRNAs 2.0	✓	✗	✗	✓	✓	✓	2021
cncRNAdb	✓	✓	✗	✓	✓	✓	2021
SPENCER	✗	✓	✓	✓	✓	✗	2022
LncPep	✓	✓	✗	✓	✓	✓	2022
LncBook 2.0	✗	✓	✓	✓	✓	✗	2023
LncSEA 2.0	✗	✓	✓	✓	✓	✗	2024

Figure S8. **The comparison of codLncDB with other databases.** Comparison of characteristics between different databases in terms of data source (Evidence), data format stored (Sequence), and involved species (Species).
